# Robust nanotube-based nanosensor designed for the detection of explosive molecules†

**DOI:** 10.1039/d4na00166d

**Published:** 2024-05-30

**Authors:** Laith A. Algharagholy, Víctor Manuel García-Suárez, Kareem Hasan Bardan

**Affiliations:** a Department of Physics, College of Science, University of Sumer Al Rifaee Zip: 64005 Thi-Qar Iraq; b Departamento de Física, Universidad de Oviedo & CINN (CSIC) Oviedo 33007 Spain garciavictor@uniovi.es

## Abstract

The adequate determination and detection of explosive molecules is key to introducing improvements in areas related to safety, whose progress depends on an adequate and rapid determination of dangerous substances. To detect explosives down to the molecular level and accurately discriminate between different but somehow similar substances, it is necessary to design sensors that can differentiate them uniquely and efficiently. In this study, we present a new generation nanoscale sensor based on carbon nanotubes with an adapted nanopore shape that is capable of effectively discriminating between five types of explosive compounds (TATP, RDX, PENT, HMX and DNT). We show that the interaction of each compound with the walls of the nanotubes induces changes in transmission and current that allows clear differentiation of each type of molecule. Interestingly, the transport properties do not depend on the orientation of the molecules within the nanopore in most cases, making it a robust device with high reproducibility and stability. The results also show that these systems can lead to relatively high thermoelectric performances and, furthermore, the Seebeck coefficient can be used to discriminate between them.

## Abstract 2

Transmissions (in a wide and narrow energy window around the Fermi level) of nanopore Tor with a TATP molecule inside and rotated 0° (a), 90° (b) 180° (c) and 270° (d).

## Graphical abstract

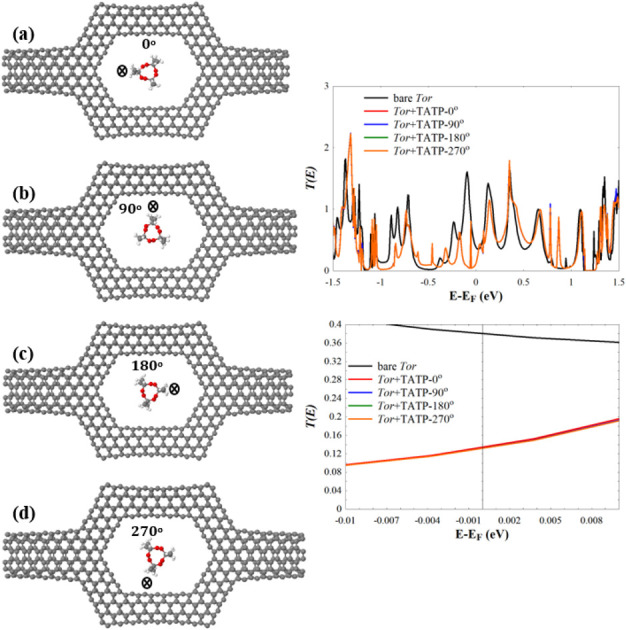

## 1. Introduction

The detection of explosives and explosive-related unlawful hazardous materials has become in recent years a significant and challenging task and has lately acquired prominence as a result of its security uses in a wide range of scenarios, including airport security screening and homeland security against terrorist threats.^1–3^ Furthermore, explosive-based compounds^4–6^ are simple to disseminate and utilize, with the potential to wreak widespread devastation. Explosives detection devices need then to be capable of detecting a wide range of explosive materials, particularly common explosives, such as triacetone triperoxide (TATP, C_9_H_18_O_6_),^7,8^ 1,3,5-trinitroperhydro-1,3,5-triazine (RDX, C_3_H_6_N_6_O_6_),^9–11^ pentaerythritol tetranitrate (PENT, C_5_H_8_N_4_O_12_),^10^ octahydro-1,3,5,7-tetranitro-1,3,5,7-tetrazocine (HMX, C_4_H_8_N_8_O_8_),^10–12^ and 2,4-dinitrotoluene (DNT, C_7_H_6_N_2_O_4_).^2,13^ To design reliable and accurate selective sensing nanodevices for highly reactive substances, there is a need to develop new nanomaterials and device concepts, along with new plans and strategies for controlling and managing nanosensor chips. Several analytical methods,^2–4,14,15^ each based on a different principle, have been employed for sensing explosive materials in the past, such as ion-mobility spectrometry,^2,16,17^ Raman spectroscopy,^18–20^ non-portable detection devices such as gas chromatography (including an electron capture detector),^4,21,22^ electronic noses,^3,23,24^ handheld mass spectrometers,^25–27^ and radiation techniques.^2–4^ There has been a significant and growing research effort^28^ devoted to develop detection methods that can further expand the versatility of the sensors, including optical, force, and electrical-based approaches. In fact, by reducing a sensor's spatial dimensions to a size comparable to those of individual molecules, single molecule sensing becomes a reality and can serve as the basis for the development of a new class termed as label-free methodology.

Label-free methods for sensing small molecules^3,29^ are desirable targets in technology because they have low costs and eliminate the need for chemical modification or separation of analytes. Nanopore technology^30^ has recently protruded as a potential method for the efficient, rapid, sensitive, and selective detection of a wide range of analytes. This field is rapidly growing and attracting a large amount of attention due to the potential applications of nanopores in many fields, such as single molecular sensing,^31,32^ genomics,^30,33,34^ biosensing,^30,33,35^ and drug discovery.^30,36,37^ Nanopore-based detection techniques^38–40^ can enable repeatable and sensitive detection down to the nanoscale (molecular level), since targeted molecules can interact with a functionalized part of the nanopore and generate thus conductivity variations that might enable single-molecule identification as well as discrimination between different species in mixture.^32^

Nanoscale nanomaterials, including heterostructure nanomaterials, might then be promising candidates for high-sensitivity chemical detectors^3,41–44^ and other nanoscale devices that have sparked intense research interest.^45,46^ Note, however, that energetic (reactive) materials such as explosive materials (compounds) that contain a great amount of potential energy, need to be handled carefully to avoid detonation.^3,47^ Experiments involving explosives, such as nitramine and aromatic explosives, present challenges.^48^ Therefore, establishment of principles and testing protocols to ensure safe manufacture and application of nanomaterials is a vital prerequisite. Despite these challenges, many experimental works^49–53^ involving explosive molecules using porous methods have been done without reported safety concerns. Furthermore, with regard to the handling of highly reactive materials, computational studies of explosives sensors can be used to complement experimental research on the design of low-power, lightweight, and inexpensive sensors.

**Fig. 1:**
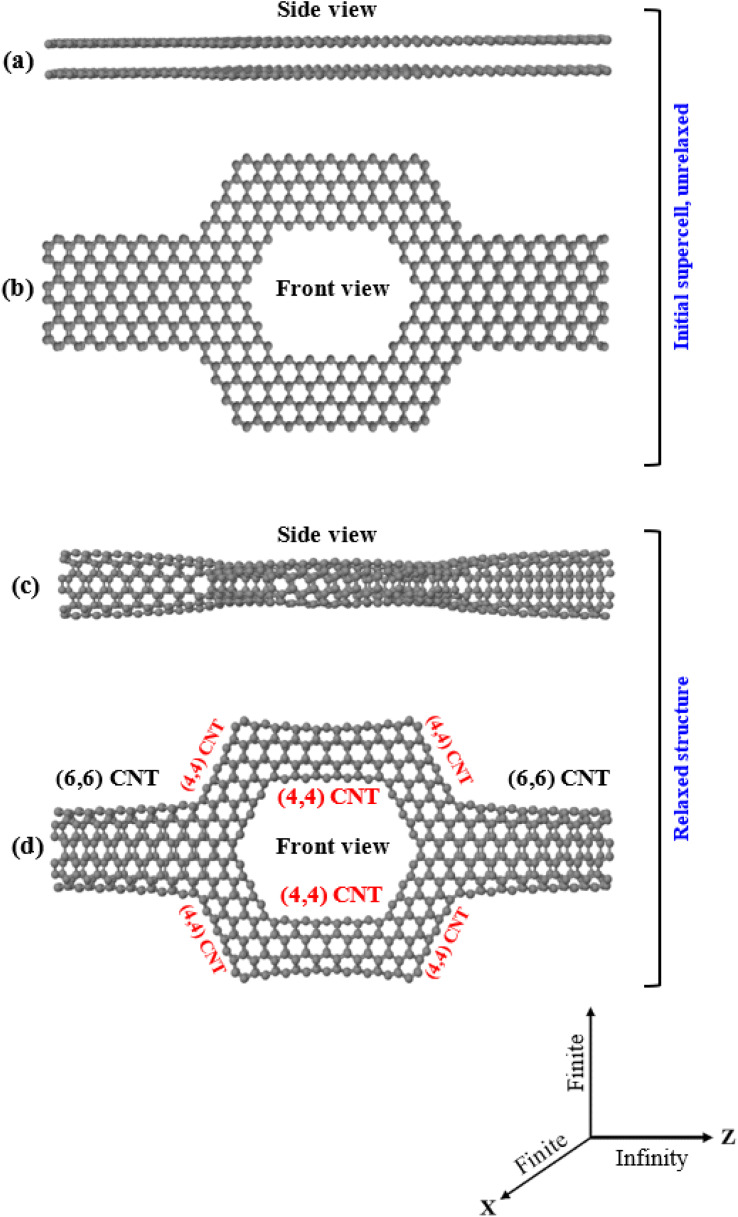
Side (a) and front (b) view of the sculpted AA-stacked bilayer graphene (initial supercell), and (c and d) side and front view of the resulting torus system (Tor), respectively. The initial supercell is periodic along the *Z* direction and finite along the *X* and *Y* directions.

In this work, we simulate a nanopore sensor designed by using carbon nanotubes, a hollow torus-like system that we refer to as Tor, shown in Fig. 1c. The proposed system consists of six (4, 4) carbon nanotubes as a scatterer (central region), whose shape is rather similar to that of a nanopore, connected to two (6, 6) carbon nanotubes that act as left and right leads. The main aim behind the design of this specific geometry based on carbon nanotubes is to present a nanosensor test concept for selective detection of a variety of explosive molecules (TATP, RDX, PENT, HMX, and DNT). When the explosive molecules pass through the central region (scattering region with a nanopore-like shape) of Tor, they interact with the carbon nanotube walls, causing changes in the potential energy of the central region locally. This interaction influences the charge density on the walls of the carbon nanotubes and leads to noticeable changes in the electronic transmission and conductance, as we shall see. The proposed system tries then to support nanopore technology and show the effectivity and reliability such method in the sensing of single molecules.

## 2. Computational tools

To obtain the optimized Tor without/with the explosive molecules shown in Fig. 1c and S1–S3,† the SIESTA^54^ implementation of density functional theory (DFT) was used. The structural optimization was carried out using the Perdew–Burke–Ernzerhof (PBE) parameterization of the generalized-gradient approximation (GGA) combined with a double-ζ polarized (DZP) basis sets of pseudoatomic orbitals and norm-conserving pseudopotentials. The real-space grid was defined with a plane-wave cut-off energy of 300 Ry, and the initial structures were optimized until the force on all atoms minimized below 0.01 eV Å^−1^. All the structures were infinite in the *Z* direction and finite in the *X* and *Y* directions, and a vacuum space of 60 Å was used along these last directions to guarantee that there is no interaction between neighboring Tor (without/with explosive molecules). Once the structures optimization was accomplished, we obtained the mean field Hamiltonian (MFH) and overlap matrices (OM) from SIESTA. Next, we fed the MFH and OM into the transport code GOLLUM,^55^ which implements equilibrium transport theory, to calculate the low-bias transmission coefficients *T*(*E*) and room-temperature current (*I*) for electrons with energy (*E*) passing through the scatterer from the left lead (source) to the right lead (drain). For the left/right leads calculations, a *k*-point grid of 1 × 1 × 25 in the Brillouin zone was used.

## 3. Characterization of Tor as a nanodevice

To obtain the hollow torus-like system (Tor) shown in Fig. 1c, we used the sculpturene method^56^ which allows to build unique and characteristic sp^2^-bonded molecular structures, such as spontaneous deterministic carbon nanotubes reconstructions out of bilayer graphene nanoribbons (bi-GNRs) or heterobilayer nanoribbons (hbi-NRs), including sp^2^-bonded T-shaped/crosslike-branched carbon nanotubes (CNT). The basic idea behind this methodology, which can also be done experimentally, is to sculpt (carve) selected/desired nanoribbons from bilayer graphene (bi-G) in vacuum. The simplest example of sculpturenes are periodic nanotubes, inholding lines of nonhexagonal rings. It is also feasible to create sculpturenes with more complex geometries formed from shapes with nontrivial topologies, connectivities and material combinations may can also be constructed.^56,57^ Cutting straight nanoribbons (NRs) from bilayer graphene (bi-G) with sufficiently small width (*i.e.* of order 3 nm or less) of the NR and allowing the edges^58,59^ to reconstruct to maximize sp^2^ bonding, the whole nanoribbon can reconstruct to form a carbon nanotube (CNT), with a predefined location and chirality. T-shaped, cross-shaped, and other multiply-connected structures are also possible to form using the sculpturene method as more complex structures with unique topologies.^56,57^ Nanomaterials can be cut using a variety of techniques, including lithographic,^60–62^ sonochemical^63,64^ and chemical^65–68^ techniques. Particularly, top/down STM lithography (STM)^62^ can be used to cut graphene nanoribbons (GNRs) with a specified chirality. In order to produce cuts with different levels of precision, specific experimental conditions are required. Since the dynamics of these techniques at the atomic scale are mostly unknown, this problem can be circumvented by beginning with bilayer graphene nanoribbons (bi-GNRs) with pre-cut edges and employing density functional theory (DFT) to enable the initial supercells to reconstruct.

We first start by sculpting or shaping AA-stacked bilayer zigzag graphene nanoribbons (bi-ZGNR) into a torus-like shape with a width of 2.840 nm (middle region) connected to bilayer zigzag graphene nanoribbons with a width of 1.136 nm (left/right regions), as shown in Fig. 1a and b. The final reconstruction of the sculpted AA-stacked bilayer graphene (Fig. 1b) leads to the formation of a hollow torus-like system (Tor), as shown in Fig. 1c, which is composed of two (6, 6) CNTs as shown in the left/right leads with well-defined ohmic contact points bonded at approximately 45° to a network of six (4, 4) carbon nanotubes (CNTs) in the region of the extended molecule (middle region, which is shaped like nanopore). Again, this process is essentially equivalent to several experimental processes,^59–61^ which have shown that folded edges can be formed by cutting bilayer/multilayer graphene. Note also that this shape is specially designed to allow target molecules to pass through it and at the same time allow interactions of them with the walls of the nanotubes that cause changes in the transport properties, as we will see. Note as well that many previous experimental works^69–79^ reported the possibility of deposition molecules/nanoparticle outside the carbon nanotube and encapsulate molecules inside the carbon nanotubes, including measurement of the conductance. Same with the nanopore, experimental works^80–83^ confirmed the ability of inserting a single molecule inside nanopores and also the measurement of the conductance, while other publications^84–86^ reported using the nanopore approach for detection of explosives.

The stability of the folded/closed structure (Fig. 1c) in comparison to the unfolded/open structure (Fig. 1a) can also be demonstrated by calculating the total energy of both cases. The energy (−129747.63 eV) is substantially lower in the folded/closed edges case than in the unfolded/open edges case (−11051.97 eV), which is expected due to the absence of unsaturated dangling bonds in the latter. The stability of Tor can also be additionally compared with that of carbon nanotubes by computing the average binding energy per atom (
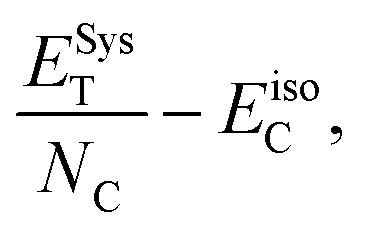
 where *E*^Sys^_T_ is the total energy of the system, *N*_C_ represents the number of carbon atoms, and *E*^iso^_C_ the energy of an isolated carbon atom). Interestingly, Tor is found to be more stable than the (4, 4) and (6, 6) CNTs, since its average binding energy (−9.89 eV) is lower than −8.67 eV and −9.76 eV for the (4, 4) and (6, 6) CNTs, respectively. Notice also that from a topological perspective, sculpturene molecular structures made of nanotubes are stable against atomic scale defects.^56^

**Fig. 2:**
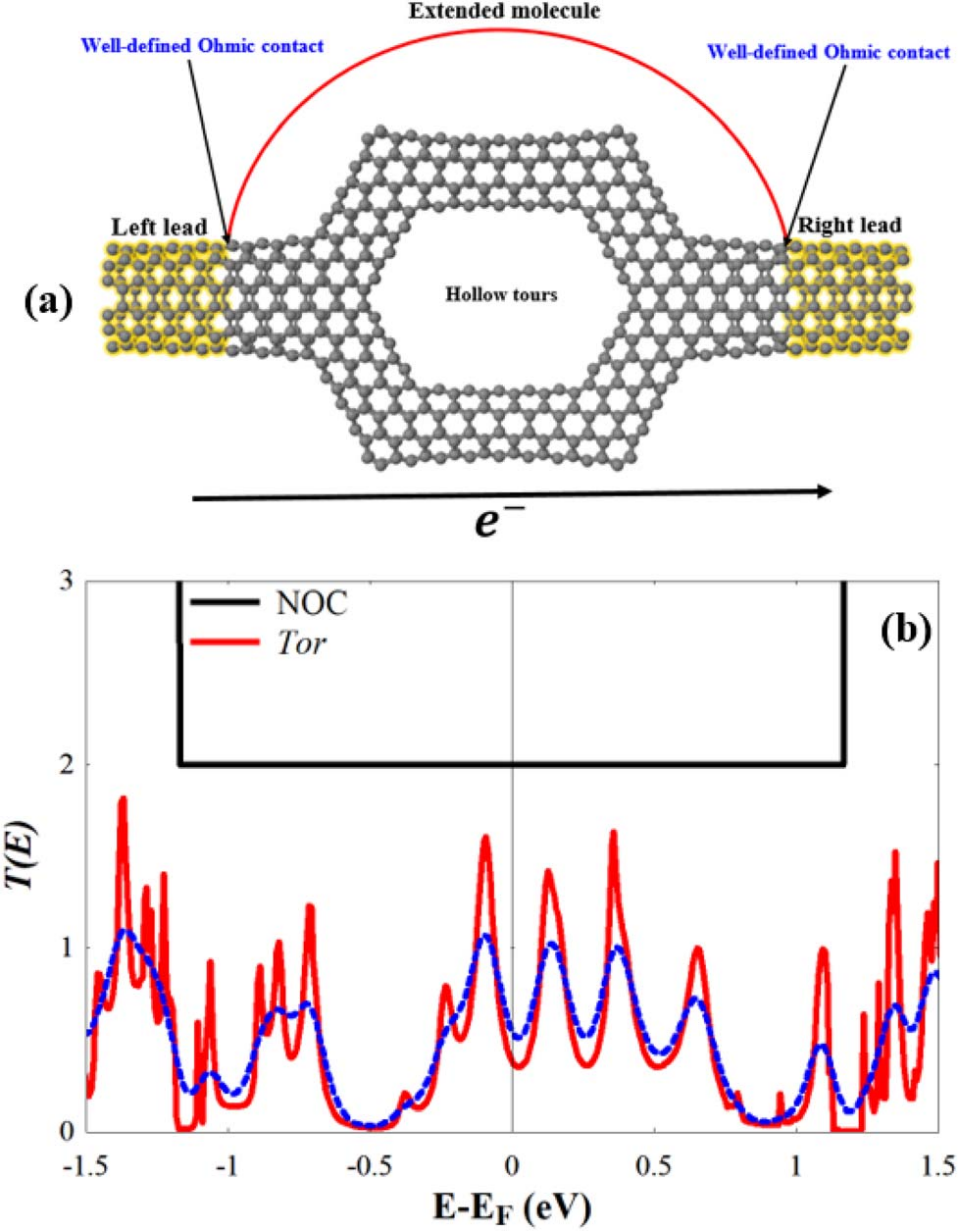
(a) Tor as a nanodevice; the yellow regions represent the left/right leads, while the grey region represents the scattering region (nanopore-like hollow torus with extended CNT). (b) *T*(*E*) of Tor; *E*_F_ is the Fermi energy value given by DFT (for convenient *E*_F_ is set to zero); the dotted blue line shows the conductance at room temperature (*T* = 300 K).

For a deeper understanding of the electronic behavior of the bare Tor shown in Fig. 2a, we investigate its transmission coefficient *T*(*E*), shown in Fig. 2b. This transmission will be used from now on as a reference to compare with Tor in contact with the explosive molecules. Despite the fact that Tor has a complex geometry made with various nanotubes, Fig. 2b shows that the *T*(*E*) of the bare Tor exhibits metallic-like properties (zero energy gap, *E*_g_) and significant values at/near the Fermi energy (*E*_F_).

## 4. Electronic transport properties of Tor with explosive molecules as nanosensor

**Fig. 3:**
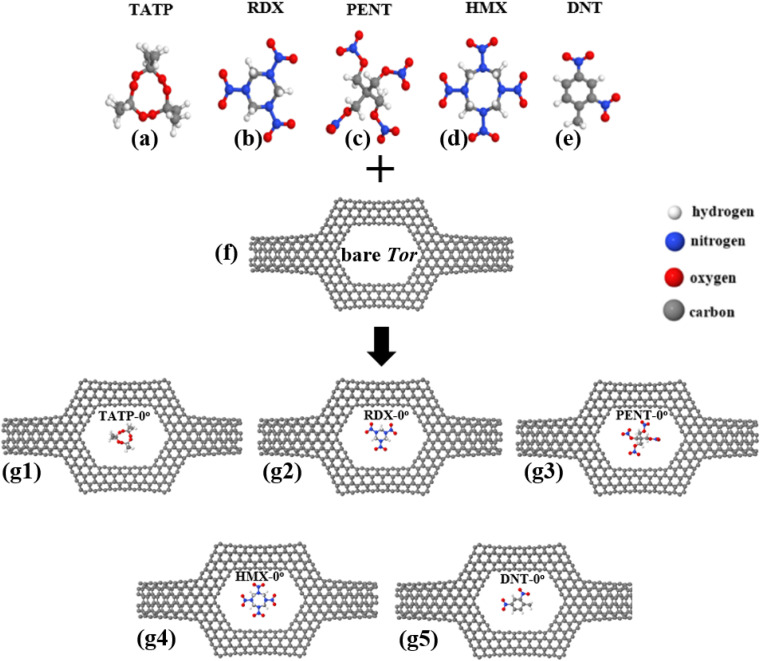
(a–e) Targeted explosive molecules TATP, RDX, PENT, HMX, and DNT respectively, (f) bare Tor, and (g1–g5) final relaxed structures of TATP, RDX, PENT, HMX, and DNT molecules inside Tor with 0° of orientation, *i.e.* Tor + TATP-0°, Tor + RDX-0°, Tor + PENT-0°, Tor + HMX-0°, and Tor + DNT-0°, respectively.

In this section, we look into the capability of Tor for selective sensing of five explosive molecules: TATP, RDX, PENT, HMX, and DNT, shown in Fig. 3a–e. Following the relaxation of Tor, the targeted explosive molecules are positioned inside it (*i.e.* inside the central region of Tor), and then the combined structures (*i.e.* Tor + TATP, Tor + RDX, Tor + PENT, Tor + HMX, and Tor + DNT) are relaxed again. Each explosive molecule is placed with four possible angle of orientation (0°, 90°, 180°, and 270°) as shown in Fig. S1–S3 of the ESI.†Fig. 3 shows the relaxed structures of the targeted explosive molecules both outside and inside Tor (with an angle of 0° in this last case). In general, after relaxation, the molecules tend to get closer to one of the edges of the pore (usually the upper edge, due to the initial unrelaxed positions). In order to have a clear idea of the position of the compounds and their distances to the wall we have also calculated the relaxed distances between their outer atoms and the nanotube walls, as can be seen in Table S1.†

We calculate first the Mulliken charges of the combined structures to determine charge transfers between the targeted molecules and Tor and get details on the electronic structure. The results are shown in Table 1. As can be seen, all *C*_T_ values are positive, which means the charge is transferred from Tor to the molecules, *i.e.* the molecules gain charge. This is to be expected since explosive molecules have more electronegative atoms (O and N) compared to Tor. The larger amount of *C*_T_ occurs for Tor + PENT and Tor + HMX in all cases, which is also anticipated given that, in comparison to TATP, RDX, and DNT molecules, PENT and HMX molecules have more electronegative atoms (O and N atoms). Since charge is transferred from the nanotube to molecule, which leads to more negatively charged nanotube walls, that implies that Tor behave as a donor. Similar charge transfers can also be observed experimentally for different molecules inside nanotubes^87^ and can serve as additional evidence of the existence of molecules within Tor.

**Table 1:** Charge transfer (*C*_T_) between Tor and the explosive molecules. The values are given in electrons per molecule

Tor	*C*_T_ (*e*)	Tor	*C*_T_ (*e*)	Tor	*C*_T_ (*e*)	Tor	*C*_T_ (*e*)
+TATP-0°	0.006	+TATP-90°	0.005	+TATP-180°	0.003	+TATP-270°	0.007
+RDX-0°	0.009	+RDX-90°	0.010	+RDX-180°	0.011	+RDX-270°	0.00
**+PENT-0°**	**0.022**	**+PENT-90°**	**0.023**	**+PENT-180°**	**0.021**	**+PENT-270°**	**0.021**
**+HMX-0°**	**0.017**	**+HMX-90°**	**0.019**	**+HMX-180°**	**0.018**	**+HMX-270°**	**0.017**
+DNT-0°	0.002	+DNT-90°	0.001	+DNT-180°	0.001	+DNT-270°	0.003

It is also imperative to ensure that the explosive molecules do not adhere to the nanotube walls throughout their passing through the scatterer (the central region). In order to check this, we calculate the binding energies (*E*_B_) for the optimized Tor with the explosive molecules using the following equation to minimise the basis set superposition error (BSSE):^88,89^

1 *E*_B_ = *E*^Tor+Mol^ − (*E*^Tor^ + *E*^Mol^)

where *E*^Tor+Mol^ is the total energy of the combined system (Tor with explosive molecule), and *E*^Tor^ and *E*^Mol^ are the total energies of the isolated Tor and explosive molecule, respectively. Table 2 shows that all binding energies are negative, which indicates that the combined system formation is exothermic and therefore can be created without additional energy. This means that, without additional energy (such as that needed to propel the molecule toward the hole and make it pass through it) the molecules would remain attached to the walls of the nanotubes, forming a stable configuration at low temperatures, and also that the molecules would be naturally favored to pass through the hole. On the other hand, the obtained binding energies are in general quite small, of the order of van der Waals energies, as expected, implying that explosive molecules will not stick to the inner walls of the carbon nanotubes in the scatterer and should pass smoothly through the hole if they are delivered through it with enough kinetic energy (similar to the typical kinetic energy of a molecule at room temperature, *i.e.* an energy enough to break the weak van der Waals bonds).

**Table 2:** Binding energies (*E*_B_) of the molecules inside the nanopore

Tor	*E*_B_ (eV)	Tor	*E*_B_ (eV)	Tor	*E*_B_ (eV)	Tor	*E*_B_ (eV)
+TATP-0°	−0.073	+TATP-90°	−0.048	+TATP-180°	−0.069	+TATP-270°	−0.069
+RDX-0°	−0.006	+RDX-90°	−0.001	+RDX-180°	−0.001	+RDX-270°	−0.001
+PENT-0°	−0.004	+PENT-90°	−0.023	+PENT-180°	−0.004	+PENT-270°	−0.027
+HMX-0°	−0.001	+HMX-90°	−0.003	+HMX-180°	−0.001	+HMX-270°	−0.003
+DNT-0°	−0.000	+DNT-90°	−0.001	+DNT-180°	−0.000	+DNT-270°	−0.015

**Fig. 4:**
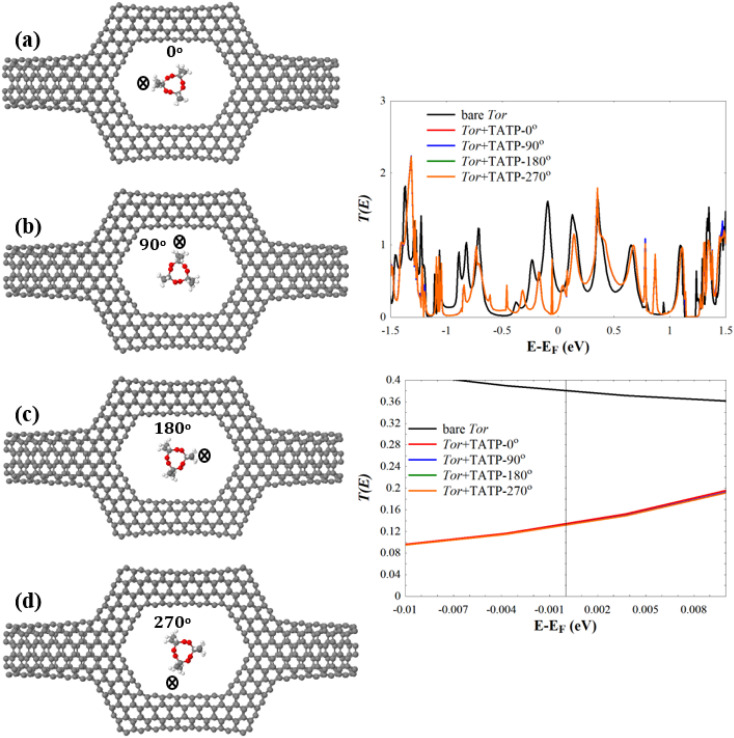
Relaxed structures of (a) Tor + TATP-0°, (b) Tor + TATP-90°, (c) Tor + TATP-180°, and (d) Tor + TATP-270°. The right subfigures show (top) *T*(*E*) of the bare Tor (black line) and the *T*(*E*) of Tor + TATP-0°, Tor + TATP-90°, Tor + TATP-180°, Tor + TATP-270°, and (bottom) *T*(*E*) in a narrow energy window (−0.01 eV to 0.01 eV) around *E*_F_.

**Fig. 5:**
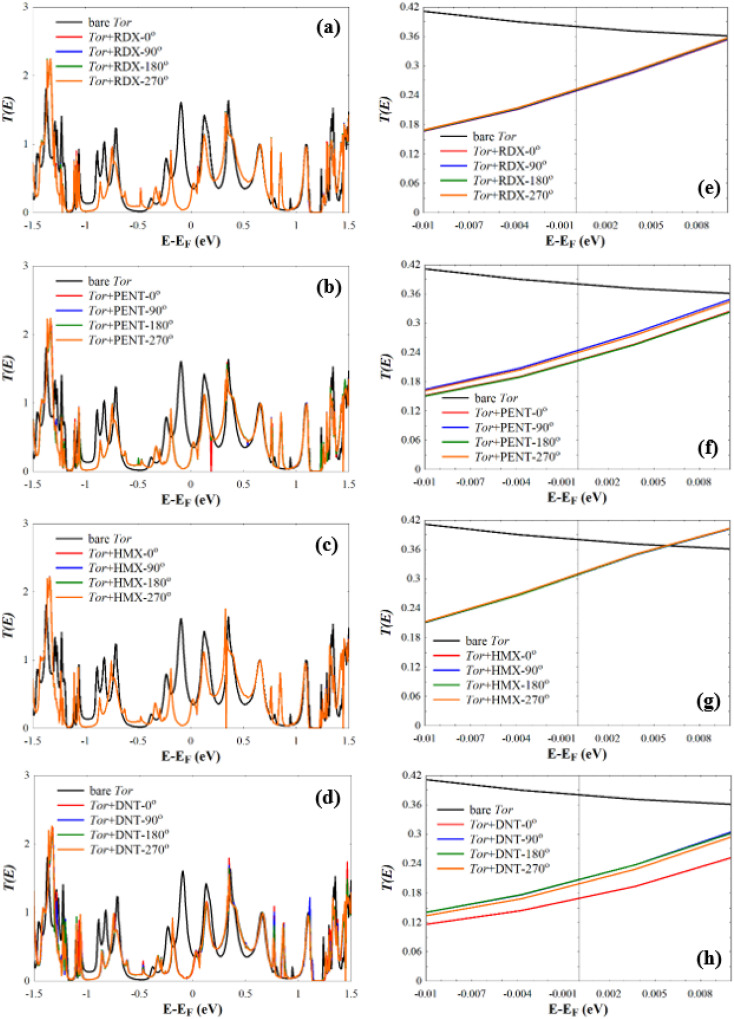
*T*(*E*) of (a) Tor + RDX-0°, Tor + RDX-90°, Tor + RDX-180°, and Tor + RDX-270°, (b) Tor + PENT-0°, Tor + PENT-90°, Tor + PENT-180°, and Tor + PENT-270°, (c) Tor + HMX-0°, Tor + HMX-90°, Tor + HMX-180°, and Tor + HMX-270°, and (d) Tor + DNT-0°, Tor + DNT-90°, Tor + DNT-180°, and Tor + DNT-270°. (e–h) *T*(*E*) shown in (a–d) in a narrow energy window (−0.01 eV to 0.01 eV) around *E*_F_.

**Fig. 6:**
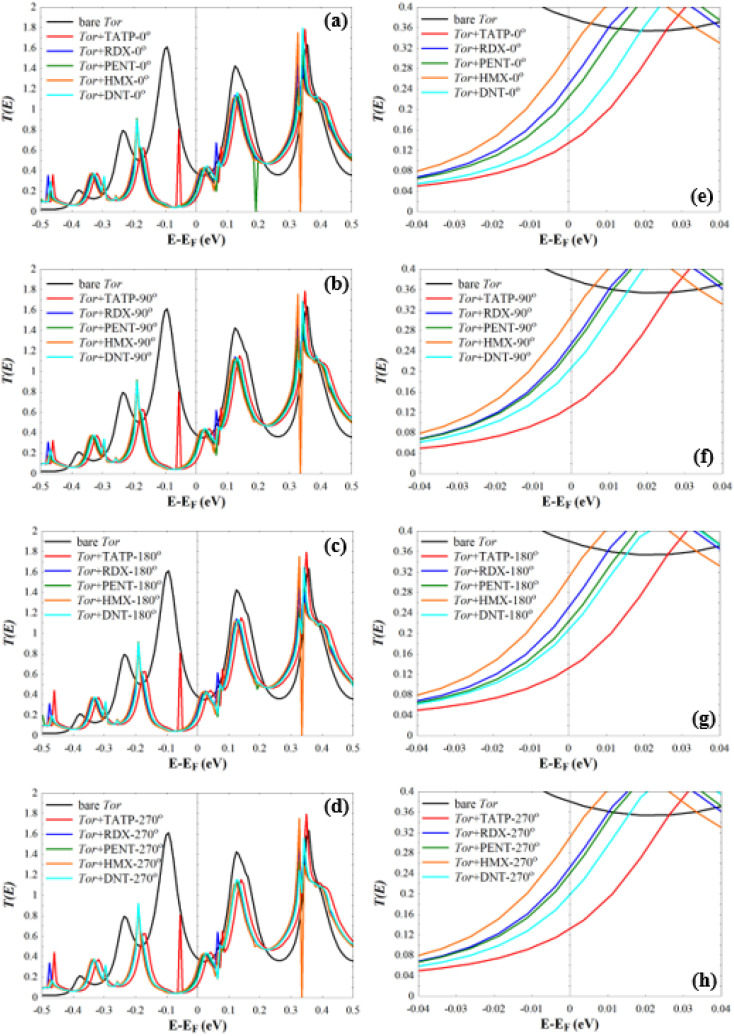
(a–d) *T*(*E*) of Tor + RDX, Tor + RDX, Tor + RDX, and Tor + RDX with 0°, 90°, 180°, and 270° of orientation, respectively. (e–h) *T*(*E*) shown in (a–d) in a narrow energy window (−0.04 eV to 0.04 eV) around *E*_F_.

Next, we investigate the low-bias transmission coefficients *T*(*E*) of Tor inside the explosive molecules. Each explosive molecule was oriented with four angles (0°, 90°, 180°, and 270°) and its transmission was compared with that of the bare Tor as a reference. Fig. 4 shows the obtained *T*(*E*) of Tor + TATP with four different angles of orientation (Tor + TATP-0°, Tor + TATP-90°, Tor + TATP-180°, Tor + TATP-270°). From Fig. 4, it is clear that *T*(*E*) does not depend on the orientation of the TATP molecule inside Tor, which means there is no angular discrimination. We repeat the same scenario for Tor + RDX, Tor + PENT, Tor + HMX, and Tor + DNT with the same four orientation angles. Again, panels Fig. 5e–h confirm that the resulting *T*(*E*) presents in general no angular discrimination, excluding somehow the cases of PENT and DNT, which for some angles have small differences. On the other hand, the results show that there is a selective sensing of the explosive molecules for each angle of orientation, since there are sizeable differences between the transmission of different ones, as shown in Fig. 6.

**Fig. 7:**
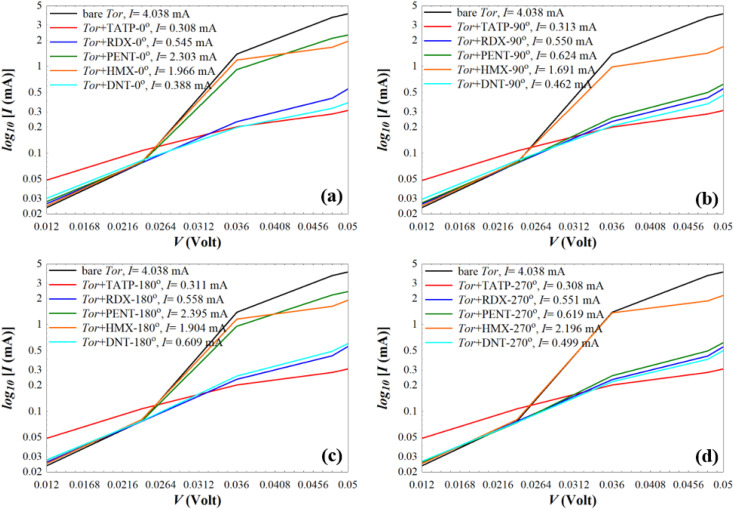
Current (*I*) of the bare Tor, Tor + TATP, Tor + RDX, Tor + PENT, Tor + HMX, and Tor + DNT with angles of (a) 0°, (b) 90°, (c) 180°, and (d) 270°.

To provide extra evidences that support the capability of Tor for selective sensing of the five targeted explosive molecules, we calculate the room-temperature current (*I*) for Tor without/with the analytes (shown in Fig. 7) using the following equation:^90,91^2
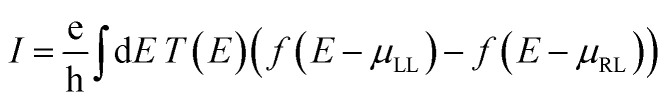
where *e* = |*e*| is the electron charge, *h* is Planck's constant, *T*(*E*) is the electronic transmission probability calculated using GOLLUM, *f* is Fermi–Dirac distribution function, and *μ*_LL_ and *μ*_RL_ are the electrochemical potentials of the left and right leads, respectively.

As can be seen from Fig. 7, for each angle orientation there is a discriminating sensing of the explosive molecules. For all cases, the current of the bare Tor at 0.05 V equals 4.038 mA, which we use as a reference guide to compare with the current of Tor with the analytes. Table S2† shows the obtained current values for all cases. Despite the fact that the differences between those values are small, previously published works^92–97^ reported that the measurement of very small currents, including nanoamperes and picoamperes, can be achieved. This means that the resulting currents shown in Table S2† and their differences can be used to selectively discriminate between the explosive molecules for each angle. Also, by comparing for each molecule the current at different angles, it is possible to assess the stability of the sensor with random equilibrium configurations. We show such comparison in Fig. S4.† As can be seen, the sensor is especially stable for the TATP and RDX molecules, which have essentially the same currents for all angles. The sensor is also rather stable for HMX and DNT, where only small differences appear for different angles. For PENT, however, which has a larger size and interacts more strongly with the walls, there are significant differences between (0°, 180°) and (90°, 270°) angles. This decreases the sensing capability for this last case, although the discriminative capability between this and the other molecules still applies. Care then should be taken when the currents of PENT are considered.

We also note that fluctuations of *T*(*E*) appearing near *E*_F_ should affect the amplitude of the Seebeck coefficient (*S*) of these systems. To show this, we computed *S* at room-temperature for Tor without/with explosive molecules as follows:^98,99^3
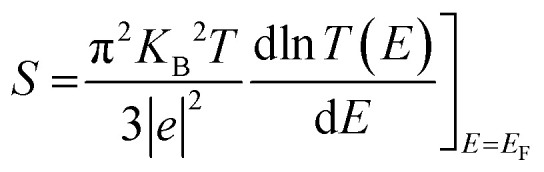


**Fig. 8:**
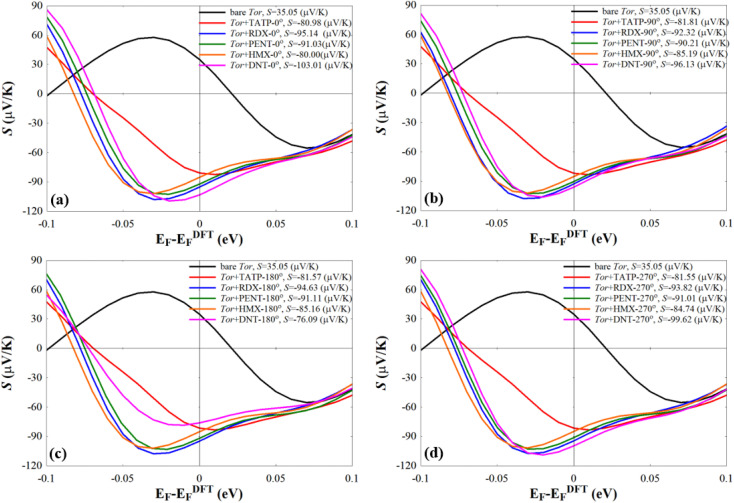
Seebeck effect (*S*) of the bare Tor, Tor + TATP, Tor + RDX, Tor + PENT, Tor + HMX, and Tor + DNT with (a) 0°, (b) 90°, (c) 180°, and (d) 270°.

Eqn (3) suggests that tuning the slope of ln *T*(*E*_F_) can enhance the value of *S*. The resulting values of *S* shown in Fig. 8 reveal that Tor (Fig. 1c) with explosive molecules inside can show a rather important increase in the value of *S* compared to that of bare Tor, *i.e.* including the explosive molecules in Tor can lead to a rather good thermoelectric performance. The differences in *S* values shown in Fig. 8 can also be utilized as additional evidence to demonstrate Tor's ability to discriminate between explosive molecules at each angle. However, more detailed research work would be needed to further demonstrate the enhancement in the thermoelectric properties of Tor, which is not the main goal of this work.

## 5. Conclusions

We have calculated the electronic, transport and thermoelectric properties of a series of explosive molecules (TATP, RDX, PENT, HMX and DNT) within a nanoscale sensor specially designed to discriminate them. The nanosensor, based on carbon nanotubes and built with the sculpturene method, is designed to allow molecules to pass through it and, at the same time, allow interactions of the molecules with the walls of the nanotubes that cause changes in transport properties. The results first show that the device (Tor) behaves as an electron donor that transfers charge to the molecules, in agreement with previous results. Furthermore, the sensor is capable, on the one hand, of producing robust and reproducible results, since the transport properties generally do not depend on the orientation of the molecules within the device, and, on the other hand, of effectively discriminating between all explosive molecules for each angle of rotation. Finally, the presence of considerable slopes near the Fermi level also allows these systems to function as efficient thermoelectric converters, also providing additional evidence to discriminate between them.

## Author contributions

Laith A. Algharagholy, Víctor Manuel García-Suárez, and Kareem Hasan Bardan were involved in interpreting the results and writing and commenting the manuscript.

## Conflicts of interest

The authors declare no conflict of interest.

## Supplementary Material

NA-006-D4NA00166D-s001
