# Supplementary material for: Robust nanotube-based nanosensor designed for the detection of explosive molecules
Source: Nanoscale Adv. 2024 May 30;6(14):3553–65. doi: 10.1039/d4na00166d (PMC11232540; doi:10.1039/d4na00166d)
Supplement: NA-006-D4NA00166D-s001 [file NA-006-D4NA00166D-s001.pdf]

Electronic Supplementary Material (ESI) for Nanoscale Advances.
This journal is © The Royal Society of Chemistry 2024

# Supplementary Information

## Robust nanotube-based nanosensor designed for the detection of explosive molecules

Laith A. Algharagholy[1], Víctor Manuel García-Suárez [2*], Kareem Hasan Bardan[1]

[1]Department of Physics, College of Science, University of Sumer, Al Rifaee, Zip: 64005, Thi-Qar, Iraq.
[2]Departamento de Física, Universidad de Oviedo & CINN (CSIC), Oviedo, 33007, Spain.
[*] Corresponding author: vm.garcia@cinn.es

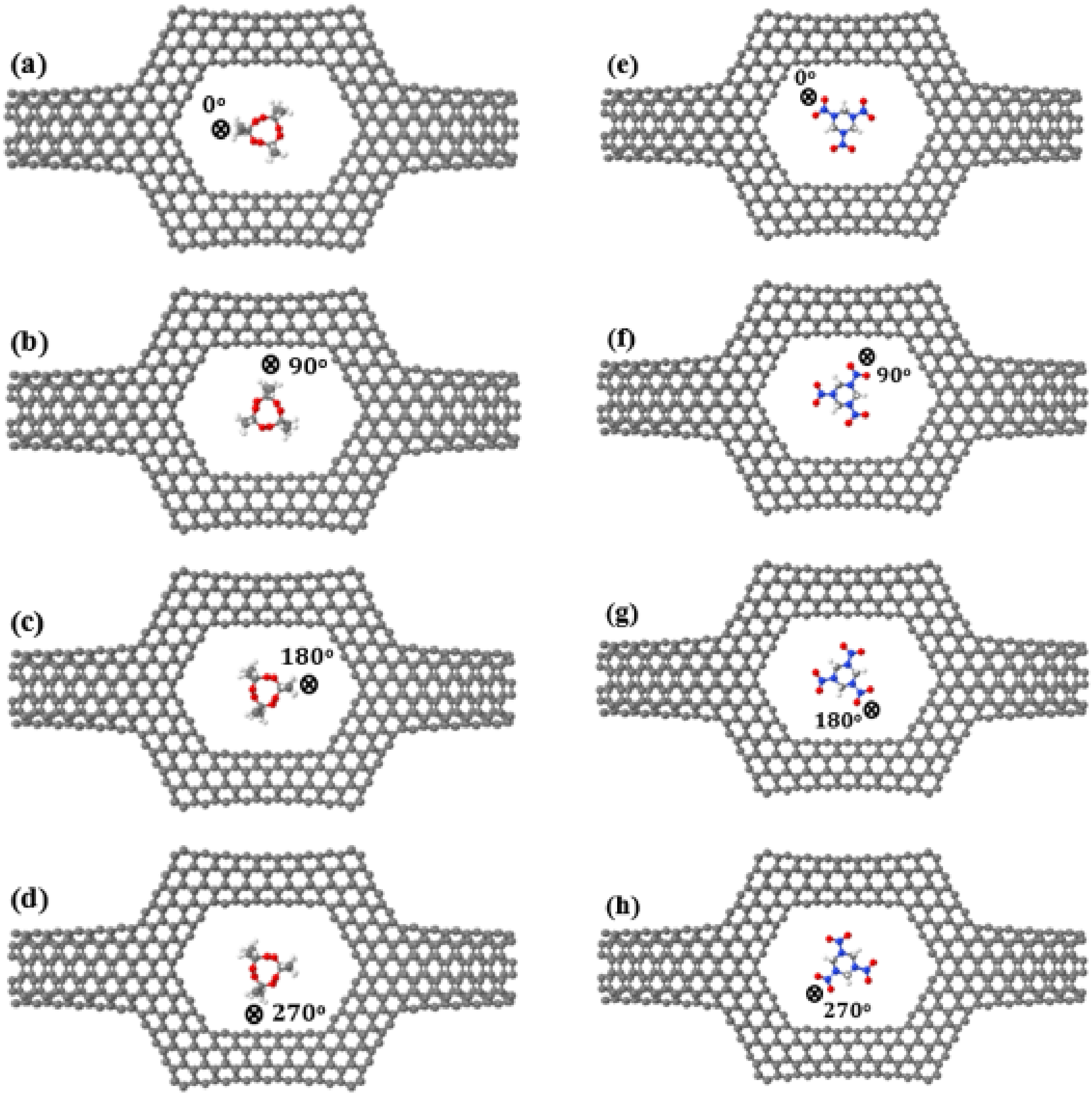


**Figure S1: (a-d)** Relaxed *Tor*+TATP with 0°, 90°, 180°, and 270°, respectively, and **(e-h)** Relaxed *Tor*+RDX with 0°, 90°, 180°, and 270°, respectively. In all subfigures, the circle with the diagonal cross symbol denotes the angle of orientation.

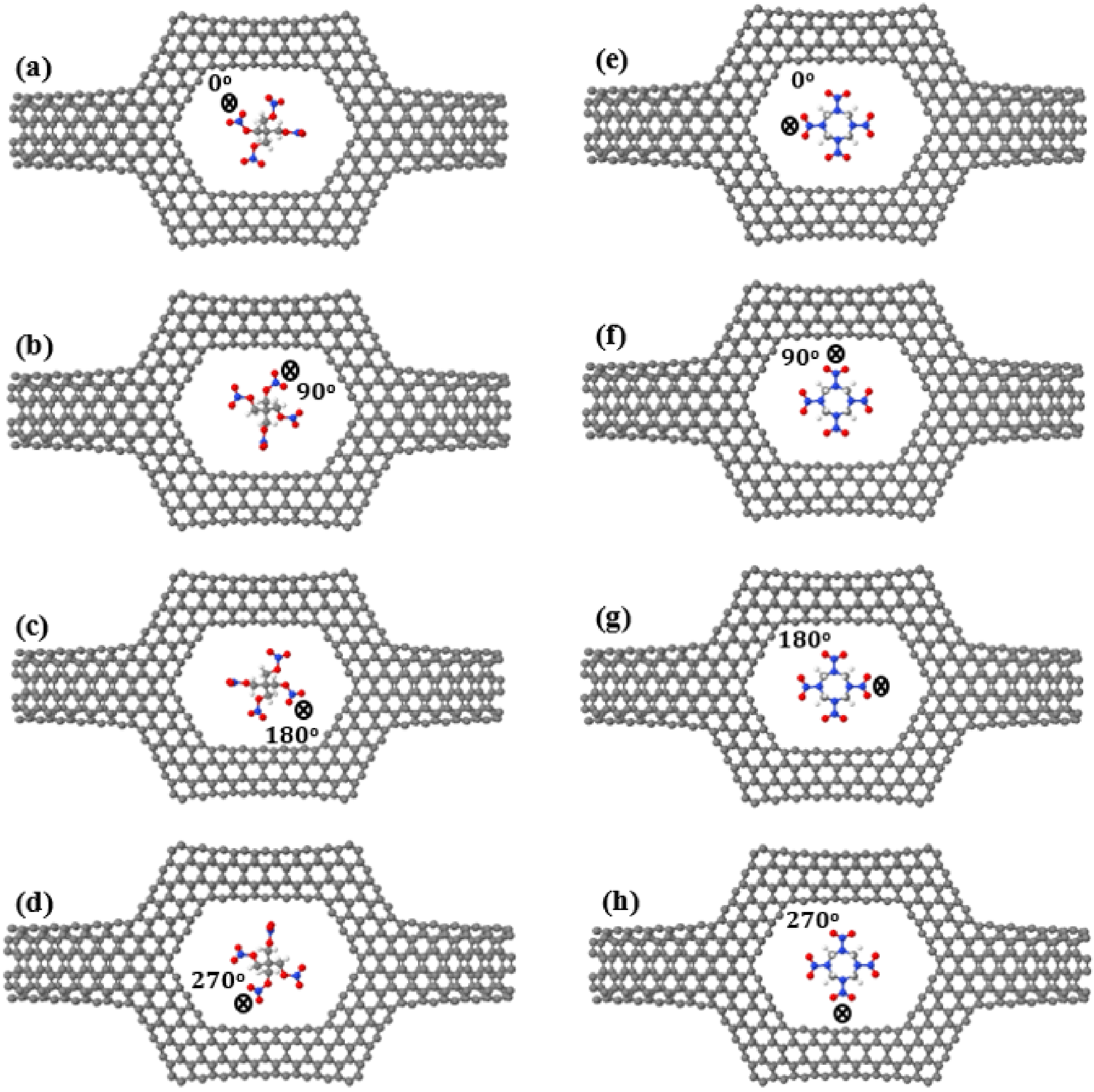


**Figure S2: (a-d)** Relaxed *Tor*+PENT with 0°, 90°, 180°, and 270°, respectively, and **(e-h)** Relaxed *Tor*+HMX with 0°, 90°, 180°, and 270°, respectively. In all subfigures, the circle with the diagonal cross symbol denotes the angle of orientation.

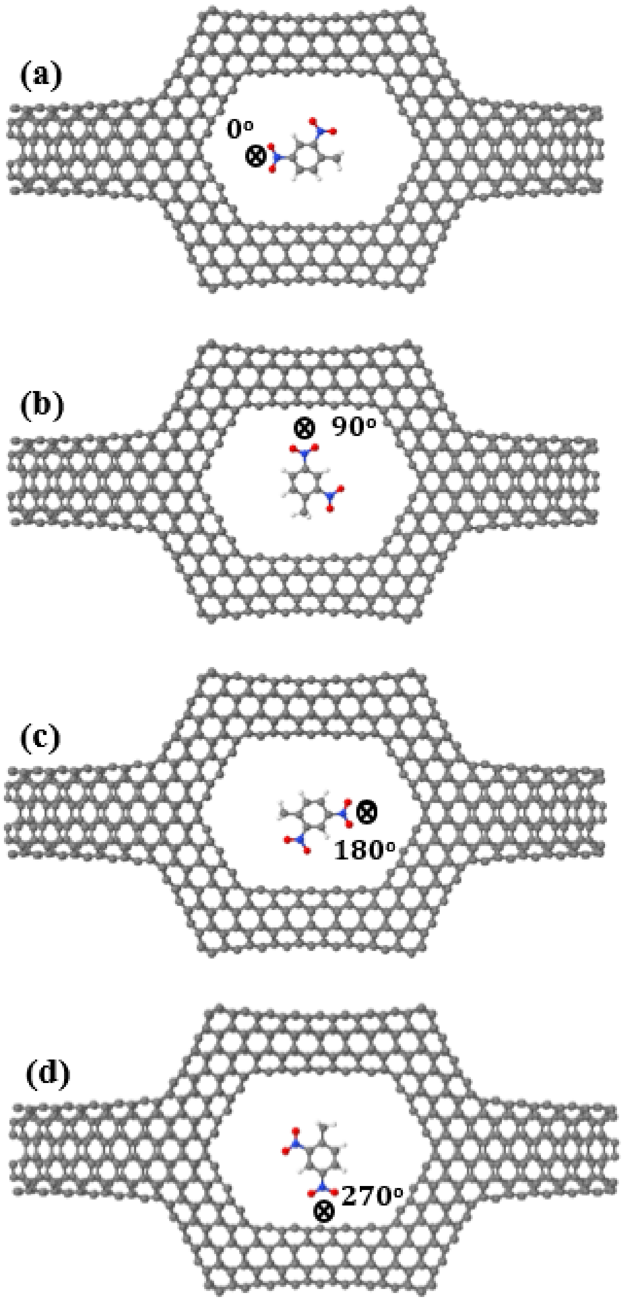


**Figure S3: (a-d)** Relaxed *Tor*+DNT with $0^{o}$, $90^{o}$, $180^{o}$, and $270^{o}$, respectively. In all subfigures, the circle with the diagonal cross symbol denotes the angle of orientation.

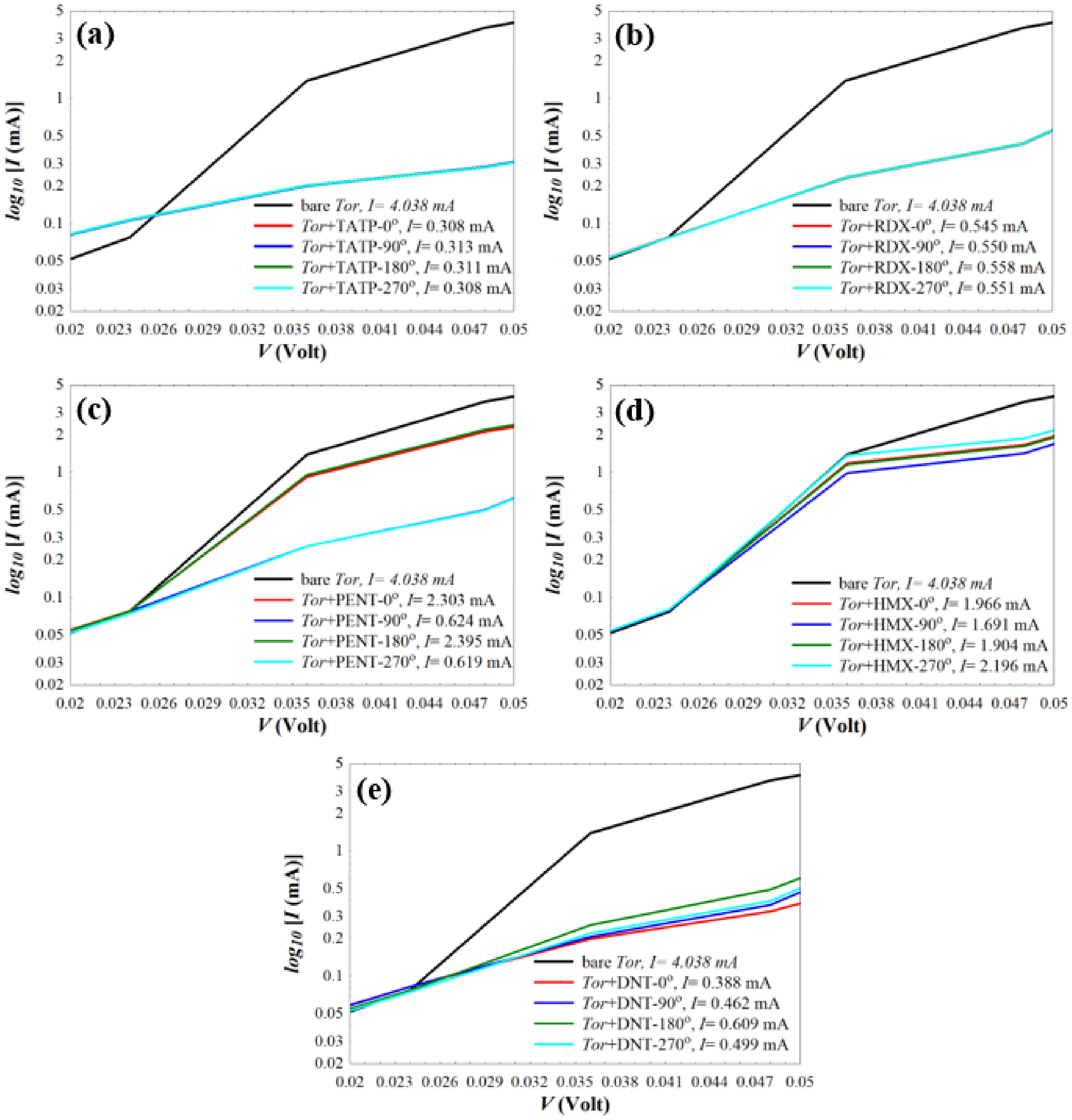


**Figure S4: (a-e)** Current (*I*) of the bare *Tor*, *Tor* +TATP, *Tor* +RDX, *Tor* +PENT, *Tor* +HMX, and *Tor*+DNT respectively. Each explosive molecule rotates with four angles of orientation inside the hollow region of the *Tor* (0°, 90°, 180°, and 270°).

**Table S1**: Distance between the inner walls of the nanopore and the closest atoms (H and O) of the explosive molecules at each orientation angle.

| | Distance in angstrom (Å) | | | |
|---|---|---|---|---|
| *Tor* | Upper edge | Lower edge | Right edge | Left edge |
| +TATP-0$^{o}$ | H= 4.84 | H= 4.41 | H= 7.57 | H= 7.15 |
| +TATP-90$^{o}$ | H= 4.97 | H= 4.52 | H= 7.36 | H= 6.99 |
| +TATP-180$^{o}$ | H= 4.80 | H= 4.12 | H= 7.38 | H= 7.29 |
| +TATP-270$^{o}$ | H= 4.68 | H= 4.13 | H= 7.84 | H= 7.27 |
| +RDX-0$^{o}$ | H= 4.99<br>O= 4.64 | H= 6.87<br>O= 4,86 | H=8 .73<br>O= 7.35 | H=7.87<br>O= 7.57 |
| +RDX-90$^{o}$ | H= 5.32<br>O= 3.95 | H= 6.13<br>O= 4.70 | H= 8.36<br>O= 7.14 | H= 8.76<br>O= 6.70 |
| +RDX-180$^{o}$ | H=5.73<br>O= 3.96 | H=6.13<br>O= 5.20 | H=8.36<br>O= 7.55 | H=8.27<br>O= 6.93 |
| +RDX-270$^{o}$ | H= 6.27<br>O= 4.05 | H= 5.69<br>O= 4.97 | H= 9.00<br>O= 7.26 | H= 7.67<br>O= 6.76 |
| +PENT-0$^{o}$ | H= 5.72<br>O= 4.15 | H= 5.64<br>O= 3.97 | H= 8.44<br>O= 5.99 | H= 9.70<br>O= 6.30 |
| +PENT-90$^{o}$ | H= 6.84<br>O= 3.70 | H= 6.79<br>O= 3.51 | H= 8.13<br>O= 6.53 | H= 8.50<br>O= 6.57 |
| +PENT-180$^{o}$ | H= 5.51<br>O= 3.92 | H= 6.13<br>O= 4.24 | H= 9.16<br>O= 5.74 | H= 9.01<br>O= 6.54 |
| +PENT-270$^{o}$ | H= 6.10<br>O= 4.44 | H= 7.00<br>O= 3.43 | H= 7.94<br>O= 6.03 | H= 8.70<br>O= 7.03 |
| +HMX-0$^{o}$ | H= 5.67<br>O= 4.13 | H= 5.57<br>O= 3.97 | H= 8.56<br>O= 6.84 | H= 8.03<br>O= 6.23 |
| +HMX-90$^{o}$ | H= 5.72<br>O= 3.93 | H= 5.55<br>O= 3.97 | H= 8.57<br>O= 6.90 | H= 8.00<br>O= 6.27 |
| +HMX-180$^{o}$ | H= 5.69<br>O= 4.10 | H= 5.56<br>O= 4.06 | H= 8.56<br>O= 6.73 | H= 8.01<br>O= 6.23 |
| +HMX-270$^{o}$ | H= 5.70<br>O= 4.07 | H= 5.55<br>O= 3.97 | H= 8.58<br>O= 6.92 | H= 7.99<br>O= 6.31 |
| +DNT-0$^{o}$ | H= 6.38<br>O= 4.94 | H= 4.78<br>O= 5.72 | H= 6.56<br>O= 8.17 | H= 8.36<br>O= 6.51 |
| +DNT-90$^{o}$ | H= 6.57<br>O= 4.45 | H= 4.15<br>O= 4.94 | H= 8.56<br>O= 7.21 | H= 7.76<br>O= 7.50 |
| +DNT-180$^{o}$ | H= 6.13<br>O= 6.76 | H= 5.84<br>O= 3.97 | H= 8.55<br>O= 6.58 | H= 7.42<br>O= 6.81 |
| +DNT-270$^{o}$ | H= 5.11<br>O= 5.92 | H= 5.59<br>O= 3.74 | H= 7.73<br>O= 7.70 | H= 7.31<br>O= 7.16 |

**Table S2:** Calculated currents at 0.05 V for all molecules inside the nanopore.

| ***Tor*** | ***I* (mA)** | ***Tor*** | ***I* (mA)** | ***Tor*** | ***I* (mA)** | ***Tor*** | ***I* (mA)** |
|---|---|---|---|---|---|---|---|
| +TATP-0$^{o}$ | 0.308 | +TATP-90$^{o}$ | 0.313 | +TATP-180$^{o}$ | 0.311 | +TATP-270$^{o}$ | 0.308 |
| +RDX-0$^{o}$ | 0.545 | +RDX-90$^{o}$ | 0.550 | +RDX-180$^{o}$ | 0.558 | +RDX-270$^{o}$ | 0.551 |
| +PENT-0$^{o}$ | 2.303 | +PENT-90$^{o}$ | 0.642 | +PENT-180$^{o}$ | 2.395 | +PENT-270$^{o}$ | 0.619 |
| +HMX-0$^{o}$ | 1.904 | +HMX-90$^{o}$ | 1.691 | +HMX-180$^{o}$ | 1.904 | +HMX-270$^{o}$ | 2.196 |
| +DNT-0$^{o}$ | 0.388 | +DNT-90$^{o}$ | 0.462 | +DNT-180$^{o}$ | 0.609 | +DNT-270$^{o}$ | 0.499 |
